# Overexpression of Indian hedgehog partially rescues short stature homeobox 2-overexpression-associated congenital dysplasia of the temporomandibular joint in mice

**DOI:** 10.3892/mmr.2015.3959

**Published:** 2015-06-18

**Authors:** XIHAI LI, WENNA LIANG, HONGZHI YE, XIAPING WENG, FAYUAN LIU, PINGDONG LIN, XIANXIANG LIU

**Affiliations:** 1 Institute of Bone Diseases, Academy of Integrative Medicine, Fujian University of Traditional Chinese Medicine, Fuzhou, Fujian 350122, P.R. China; 2 Research Base of Traditional Chinese Medicine Syndrome, College of Traditional Chinese Medicine, Fujian University of Traditional Chinese Medicine, Fuzhou, Fujian 350122, P.R. China; 3 Fujian Key Laboratory of Integrative Medicine on Geriatrics, Fujian University of Traditional Chinese Medicine, Fuzhou, Fujian 350122, P.R. China

**Keywords:** temporomandibular joint, extracellular matrix, matrix metalloproteinase, Indian hedgehog

## Abstract

The role of short stature homeobox 2 (shox2) in the development and homeostasis of the temporomandibular joint (TMJ) has been well documented. Shox2 is known to be expressed in the progenitor cells and perichondrium of the developing condyle. A previous study by our group reported that overexpression of shox2 leads to congenital dysplasia of the TMJ via downregulation of the Indian hedgehog (Ihh) signaling pathway, which is essential for embryonic disc primordium formation and mandibular condylar growth. To determine whether overexpression of Ihh may rescue the overexpression of shox2 leading to congenital dysplasia of the TMJ, a mouse model in which Ihh and shox2 were overexpressed (*Wnt1-Cre*; *pMes-stop shox2*; *pMes-stop Ihh mice*) was utilized to assess the consequences of this overexpression on TMJ development during post-natal life. The results showed that the developmental process and expression levels of runt-related transcription factor 2 and sex determining region Y-box 9 in the TMJ of the *Wnt1-Cre*; *pMes-stop shox2*; *pMes-stop Ihh* mice were similar to those in wild-type mice. Overexpression of Ihh rescued shox2 overexpression-associated reduction of extracellular matrix components. However, overexpression of Ihh did not inhibit the shox2 overexpression-associated increase of matrix metalloproteinases (MMPs) MMP9, MMP13 and apoptosis in the TMJ. These combinatory cellular and molecular defects appeared to account for the observed congenital dysplasia of TMJ, suggesting that overexpression of Ihh partially rescued *shox2* overexpression-associated congenital dysplasia of the TMJ in mice.

## Introduction

The temporomandibular joint (TMJ), a highly specialized synovial joint, permits movement and function of the mammalian jaw, which consists of a glenoid fossa, a condylar head of the mandible and a fibrocartilaginous disc (1,2). The glenoid fossa as well as the condyle develop from two distinct mesenchymal condensations, the temporal and condylar blastemas, and ossify through specific mechanisms: The glenoid fossa undergoes intramembranous ossification, while the condyle is endochondral in origin (3,4). Although TMJ development during embryonic and post-natal life involves complex processes that are well documented, little information is available regarding the cellular and molecular mechanisms involved in TMJ morphogenesis.

The condyle is a crucial growth site in the mandible and displays similarities with the growth plate of the long bones. It has four distinct zones, including a zone of hypertrophic chondrocytes, a zone of flattened chondrocytes, a progenitor cell layer and a fibrous cell layer (5). One key gene which was previously reported to be expressed in the growth plate of the condylar cartilage is Indian hedgehog (Ihh), which belongs to a family of potent secreted signaling proteins (6). Ihh is critical for maintaining the growth of adjacent proliferating chondrocytes and has an indirect role in controlling the rate of chondrocyte differentiation by acting in a negative feedback loop with parathyroid-hormone-related protein (PTHrP) in the periarticular perichondrium (7,8). Ihh, in conjunction with PTHrP, is essential for organizing the growth plate (9). Ihh acts on target cells through the cell-surface receptors Smoothened (Smo) and Patched (Ptc) and in cooperation with primary cilia, and signaling is mediated by the Gli family of transcription factors (10–12). Gli1, a transcriptional activator of Ihh targets, is transcriptionally upregulated by Ihh signaling. In mice, cartilage development begins in the center of the condyle, and Ihh begins to have a significant effect in the condylar cartilage at E15.5 (6,13). In Ihh-/- mutant mice, the organization of the growth-plate-like zone in the condyle is disrupted, and the articular disc does not form (6). In summary, Ihh is crucial for TMJ formation and development, where it appears to regulate the condylar cartilage phenotype, chondroprogenitor cell function, as well as growth and elongation events.

A previous study by our group showed that downregulation of the Ihh signaling pathway is one of the causes of congenital dysplasia of the TMJ in mice with short stature homeobox 2 (shox2) overexpression (14). Shox2, a member of shox gene family, is found only in vertebrates, suggesting its role in the development of the internal skeleton and its associated structures (4,13,14). The present study was conducted to determine whether overexpression of Ihh may rescue the shox2 overexpression-associated effects on the TMJ phenotype using a mouse model of Ihh and shox2 overexpression (*Wnt1-Cre*; *pMes-stop shox2*; *pMes-stop Ihh* mice).

## Materials and methods

### Mouse embryo collection

The animal procedure used in the present study was approved by the Institutional Animal Care and Use Committee (IACUC) of Fujian University of Traditional Chinese Medicine (Fuzhou, China). A total of 72 *Wnt1-Cre*; *pMes-stop shox2*; *pMes-stop Ihh* mice, generated by crossing *Wnt1-Cre*; *pMes-stop shox2* mice (14) with *pMes-stop Ihh* mice, and a total of 72 wild-type mice were provided by the laboratory of Dr Yiping Chen (Department of Cell and Molecular Biology, Tulane University, New Orleans, LA, USA). The mice were exposed to a 12 h light/dark cycle in a temperature (22±1°C) and humidity (56±1%) controlled environment. They were maintained on a 0.3% sodium diet and were housed five/cage. The mice were sacrificed with CO_2_ and the embryos were extracted in phosphate-buffered saline (PBS; pH 7.4; GE Healthcare Life Sciences; Logan, UT, USA) at 4°C. When the presence of a vaginal plug was found in the morning, the age of the embryo was defined as embryonic day 0.5 (E0.5). Embryonic heads were fixed in 4% paraformaldehyde (PFA) (Sigma-Aldrich, St Louis, MO, USA)/PBS at 4°C overnight. The heads of post-natal day 0 (P0), P7, P14 and P21 mice were fixed and decalcified in Surgipath Decalcifier I (Leica Biosystems Richmond Inc., Richmond, CA, USA) for different times depending on the age of the mouse according to the manufacturer's instructions (4).

### Histological analyses

Paraffin-embedded heads were sectioned at a thickness of 10 *µ*m with a microtome. For histological analysis of the TMJ, the serial sections were stained with azon red/anilin blue staining (Sigma-Aldrich) according to standard procedures. Briefly, paraffin sections were deparaffinizated in xylene (Sigma-Aldrich) and rehydrated through a gradient series of ethanol concentrations (Merck & Co., Inc., Kenilworth, NJ, USA). They were then stained with 0.5% azon red (Sigma-Aldrich) for 2 h at 56°C, rinsed in 5% phospho-tungstic acid (Sigma-Aldrich) for 3 h at room temperature, stained with 0.5% anilin blue (Sigma-Aldrich) for 2 h, then were dehydrated and mounted on slides. The morphology of TMJ was observed under an Olympus BH-2 light microscope (Olympus, Tokyo, Japan).

### Immunohistochemistry

Paraffin-embedded heads were sectioned at a thickness of 8 *µ*m for immunohistochemistry. Subsequent to blocking with goat serum (1:10; Invitrogen Life Technologies, Carlsbad, CA, USA) and incubation for 15 min at room temperature, the sections were incubated with polyclonal antibodies against runt-related transcription factor 2 (Runx2; 1:1,000; ab76956), sex determining region Y-box 9 (Sox9; 1:500; ab26414), collagen type I (col I) (1:500; ab34710), collagen type II (col II) (1:200; ab53047), aggrecan (1:500; ab36861), matrix metalloproteinase 9 (MMP9) (1:300; ab38898), MMP13 (1:50; ab75606) and Ihh (1:200; ab39634) from Abcam (Cambridge, MA, USA) overnight at 4°C. The slides were then washed three times with PBS and were incubated with a biotinylated horseradish peroxidase goat anti-rabbit secondary antibody (1:1,000 dilution; Invitrogen Life Technologies) for 20 min at 37°C. The slides were then washed three times with the secondary antibody using PBS. Immunolabelling was visualized with 0.05% diaminobenzidine (Invitrogen Life Technologies) in PBS for 5 min at room temperature, and the slides were then rinsed for 10 min under running tap water. The TMJ immunohistochemical staining was analyzed under the Olympus BH-2 light microscope.

### In situ zymography

Heads of P0 mice were fixed in zinc-based fixative (ZBF; 36.7 mM ZnCl, 27.3 mM ZnAc_2_ × 2H_2_O and 0.63 mM CaAc in 0.1 mM Tris pH 7.4; all from Sigma-Aldrich) and frozen in optimum cutting temperature compound (Tissue-Tek; Sakura Finetek USA, Inc., Torrance, CA, USA) compound using liquid nitrogen. According to the manufacturer's instructions, 10-*µ*m sections were incubated at 37°C for 2 h in a dark humidified chamber with 1 mg/ml dye-quenched gelatin (100 *µ*l; E12055; Molecular Probes Europe BV, Leiden, The Netherlands), which was applied on top of the sections and was covered with a cover-slip (14–17). The gelatinolytic activity was observed as green fluorescence under a fluorescence microscope (Axioskop 50; CarlZeiss, Oberkochen, Germany).

### Terminal deoxynucleotidyl transferase dUTP nick end labeling (TUNEL) assay and bromodeoxyuridine (BrdU) labeling assay

Apoptosis was tested using the *In Situ* Cell Death Detection kit, alkaline phosphatase (Roche, Basel, Switzerland). Paraffin-embedded heads were sectioned at 5 *µ*m and subjected to immunodetection according to the manufacturer's instructions (4,14). At E14.5 or E16.5, pregnant mice were injected with 1.5 ml labeling reagent/100 g using the BrdU Labeling and Detection Kit II (Roche) for 2 h. The heads of embryonic mice were fixed in Carnoy's fixative [6:3:1 ratio of ethanol (Merck & Co., Inc.), chloroform (Sigma-Aldrich) and glacial acetic acid (Merck & Co., Inc.)], ethanol-dehydrated, paraffin-embedded and sectioned at 5 *µ*m. The sections were subjected to immunodetection for analysis of cell proliferation according to the manufacturer's instructions (13).

### Statistical analysis

All experiments were repeated at least three times, and values are expressed as the mean ± standard deviation. Statistical analyses of the data were performed using the Student's t-test with SPSS software, version 13.0 (SPSS, Inc., Chicago, IL, USA). P<0.05 was considered to indicate a statistically significant difference.

## Results

### Wnt1-Cre; pMes-stop shox2; pMes-stop Ihh mice exhibit TMJ dysplasia

**Figure 1:**
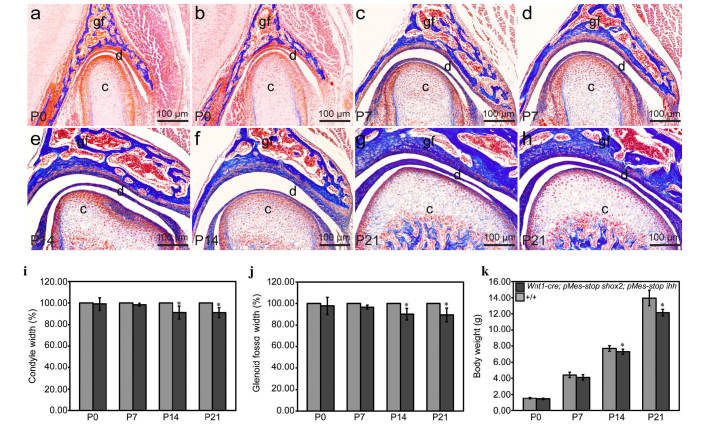
Histological analyses of TMJs of *Wnt1-Cre*; *pMes-stop shox2*; *pMes-stop Ihh* mice. (a-h) Azon red/anilin blue-stained coronal sections of the TMJ in wild-type mice and *Wnt1-Cre*; *pMes-stop shox2*; *pMes-stop Ihh* mice, respectively, at (a and b) P0, (c and d) P7, (e and f) P14 and (g and h) P21. (i and j) Comparison of the condyle and glenoid fossa width in wild-ype mice and *Wnt1-Cre*; *pMes-stop shox2*; *pMes-stop Ihh* mice at different stages. (k) Comparison of body weight at various time-points in wild-type mice and *Wnt1-Cre*; *pMes-stop shox2*; *pMes-stop Ihh* mice. Values are expressed as the mean ± standard deviation (n=3). ^*^P<0.05 vs. wild-type. gf, glenoid fossa; d, disc; c, condyle; TMJ, temporomandibular joint; P, post-natal day.

Histological analyses of TMJs did not indicate any differences between wild-type mice and *Wnt1-Cre*; *pMes-stop shox2*; *pMes-stop Ihh* mice at P0 and P7, while TMJ dysplasia was observed at P14. To investigate the phenotype of the TMJs, a time-course analysis of alternation in the width of the glenoid fossa and condyle of the TMJ was performed. The observation was focused on the widest part of the glenoid fossa and condyle where the defect was most significant. The average width (obtained from three measurements of different animals) of the glenoid fossa and condyle in the wild-type mice at each time-point was defined as 100%. That the width of the glenoid fossa and condyle appeared to be similar between wild-type mice and *Wnt1-Cre*; *pMes-stop shox2*; *pMes-stop Ihh* mice at P0 and P7 (Fig. 1a–d). However, at P14 and P21, the width of the condyle and glenoid fossa in the *Wnt1-Cre*; *pMes-stop shox2*; *pMes-stop Ihh* mice was decreased compared with that in the wild-type mice (P<0.05) (Fig. 1e–j). These results suggested that the TMJ dysplasia occurred primarily in the second week following birth.

To assess the effect of the TMJ dysplasia on the body weight in the *Wnt1-Cre*; *pMes-stop Shox2*; *pMes-stop Ihh* mice, the body weight was determined at different developmental stages (P0, P7, P14 and P21) (Fig. 1k). Mice stopped suckling and started eating solid food at P21. The results demonstrated that the body weight between wild-type mice and *Wnt1-Cre*; *pMes-stop shox2*; *pMes-stop Ihh* mice was similar at P0 and P7; however, it was significantly different at P14 and P21 (P<0.05), indicating that the observed changes of the TMJ in *Wnt1-Cre*; *pMes-stop shox2*; *pMes-stop Ihh* mice may have affected the function of the TMJ, leading to a reduction in body weight and the development of a wasting syndrome.

### Wnt1-Cre; pMes-stop shox2; pMes-stop Ihh mice display normal expression of Runx2 and Sox9 in the developing TMJ

**Figure 2:**
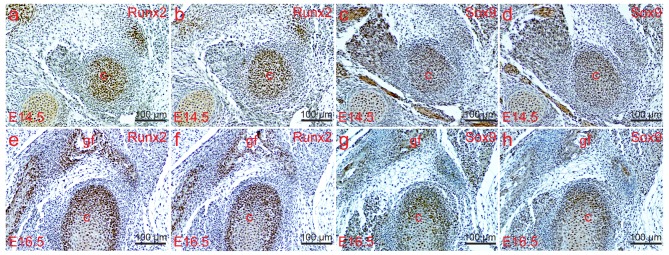
Expression of Runx2 and Sox9 in the developing temporomandibular joint of *Wnt1-Cre*; *pMes-stop shox2*; *pMes-stop Ihh* mice. (a–h) Immunohistochemical staining shows Runx2 and Sox9 expression in the developing condyle of (a-d) E14.5 and (e-h) E16.5 wild-type mice and *Wnt1-Cre*; *pMes-stop shox2*; *pMes-stop Ihh* mice, respectively. gf, glenoid fossa; c, condyle; E, embryonic day; Runx2, runt-related transcription factor 2; Sox9, sex determining region Y-box 9.

To determine whether the protein levels of Runx2 and Sox9 were altered in the developing TMJ of *Wnt1-Cre*; *pMes-stop shox2*; *pMes-stop Ihh* mice, immunohistochemical analysis was performed. There were no differences in Runx2 and Sox9 protein levels in the TMJ between wild-type mice and *Wnt1-Cre*; *pMes-stop shox2*; *pMes-stop Ihh* mice at E14.5 and E16.5 (Fig. 2a–h), suggesting that overexpression of Ihh did not affect the formation and development of the TMJ during the embryonic stage.

### Wnt1-Cre; pMes-stop shox2; pMes-stop Ihh mice show no abnormalities in the extracellular matrix (ECM) composition

**Figure 3:**
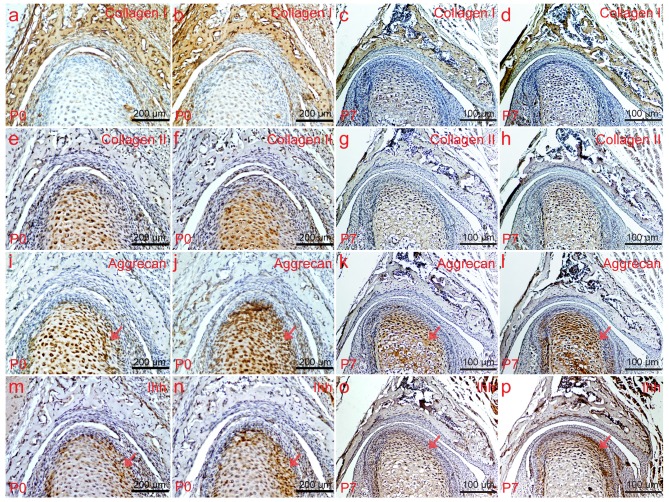
Protein levels of extracellular matrix and Ihh in the TMJ of post-natal *Wnt1-Cre*; *pMes-stop shox2*; *pMes-stop Ihh* mice. (a-l) Protein levels of (a-d) col I, (e-h) col II, (i-l) aggrecan and (m-p) Ihh in the TMJ of P0 and P7 in wild-type mice and *Wnt1-Cre*; *pMes-stop shox2*; *pMes-stop Ihh* mice, respectively. The red arrows indicate the condyle where the expression is altered. Ihh, Indian hedgehog; TMJ, temporomandibular joint; P, post-natal day; col, collagen.

The ECM, including collagens, proteoglycans and glycosaminoglycans, is the main component of articular cartilage (4). It is essential for the resistance to compressive forces and maintaining the tensile properties of the cartilage. A previous study by our group showed that overexpression of shox2 led to a reduction of col I and col II expression (14). To account for the dysplasia of TMJ in the *Wnt1-Cre*; *pMes-stop shox2*; *pMes-stop Ihh* mice, the expression of several key components of the ECM, including col I, col II and aggrecan was assessed at P0 and P7 (Fig. 3a–l). Immunohistochemcal analysis showed that the protein levels of col I and col II were similar between wild-type and *Wnt1-Cre*; *pMes-stop shox2*; *pMes-stop Ihh* mice at P0 and P7. However, the protein levels of aggrecan were increased in the condyle in the TMJ of *Wnt1-Cre*; *pMes-stop shox2*; *pMes-stop Ihh* mice. These results suggested that overexpression of Ihh rescued the ECM composition in the *Wnt1-Cre*; *pMes-stop shox2* mice.

### Ihh is overexpressed in the TMJ of Wnt1-Cre; pMes-stop shox2; pMes-stop Ihh mice

To determine whether the expression of Ihh was significantly upregulated in the developing TMJ of *Wnt1-Cre*; *pMes-stop shox2*; *pMes-stop Ihh* mice, the protein levels of Ihh were assessed using immunohistochemistry at P0 and P7 (Fig. 3m–p). The protein levels of Ihh were increased in the condyle of *Wnt1-Cre*; *pMes-stop shox2*; *pMes-stop Ihh* mice compared with those in the wild-type mice, supporting that Ihh was overexpressed in the TMJ.

### MMPs activity is upregulated in the TMJ of Wnt1-Cre; pMes-stop shox2; pMes-stop Ihh mice

**Figure 4:**
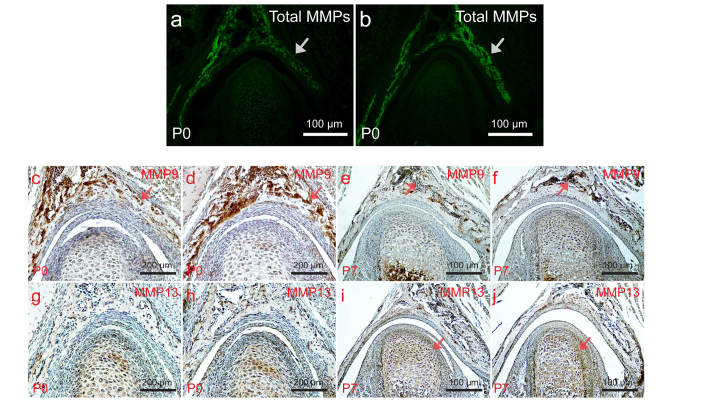
Altered expression of MMPs in the TMJs of post-natal *Wnt1-Cre*; *pMes-stop shox2*; *pMes-stop Ihh* mice. (a and b) *In situ* zymography of coronal sections of the TMJ in (a) wild-type mice and (b) *Wnt1-Cre*; *pMes-stop shox2*; *pMes-stop Ihh* mice was performed to assess MMP activity (indicated by arrow heads). (c–j) Immunohistochemical staining for (c-f) MMP9 and (g–j) MMP13 of coronal sections from (c, d, g, i) wild-type mice and (d, f, h, j) *Wnt1-Cre*; *pMes-stop shox2*; *pMes-stop Ihh* mice (arrow heads). MMP, matrix metalloproteinase; TMJ, temporomandibular joint; P, post-natal day.

The previous study by our group showed that overexpression of shox2 results in the upregulation of MMP activity (14). In the present study, it was therefore assessed whether overexpression of Ihh is able to inhibit the enhancement of MMP activity. First, *in situ* zymography was performed to determine total MMP activity in the TMJs of wild-type mice and *Wnt1-Cre*; *pMes-stop shox2*; *pMes-stop Ihh* mice at P0. As shown in Fig. 4a and b, enhanced MMP activity was identified in the glenoid fossa; however, above-background levels of MMP activity were also observed in the condyle. Consistent with this enhanced total MMP activity, the expression of MMP9 and MMP13 in the TMJs of *Wnt1-Cre*; *pMes-stop shox2*; *pMes-stop Ihh* mice was found to be enhanced compared with that in the wild-type mice at P0 and P7 (Fig. 4c–j). These results indicated that enhanced MMP activity is associated with dysplasia of the TMJ, and that the shox2 overexpression-associated increases in MMP activity was not sufficiently rescued by overexpression of Ihh.

### Apoptosis is enhanced in the glenoid fossa of the Wnt1-Cre; pMes-stop shox2; pMes-stop Ihh mice

**Figure 5:**
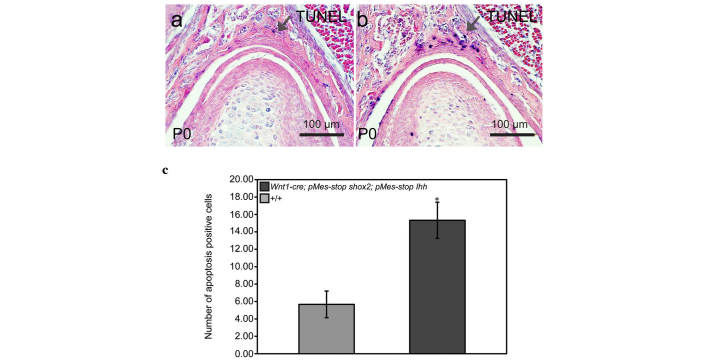
Altered apoptosis in the TMJs of post-natal *Wnt1-Cre*; *pMes-stop shox2*; *pMes-stop Ihh* mice. (a and b) TUNEL assay on coronal sections of the TMJs in (a) wild-type mice and (b) *Wnt1-Cre*; *pMes-stop shox2*; *pMes-stop Ihh* mice shows an increase of apoptotic cells in the glenoid fossa of the *Wnt1-Cre*; *pMes-stop shox2*; *pMes-stop Ihh* mice (arrow heads). (c) Comparison of numbers of apoptotic cells in the glenoid fossa between wild-type mice and *Wnt1-Cre*; *pMes-stop shox2*; *pMes-stop Ihh* mice. Values are expressed as the mean ± standard deviation (n=3). ^*^P<0.05 vs. wild-type. TMJ, temporomandibular joint; P, post-natal day; TUNEL, terminal deoxynucleotidyl transferase dUTP nick end labeling.

**Figure 6:**
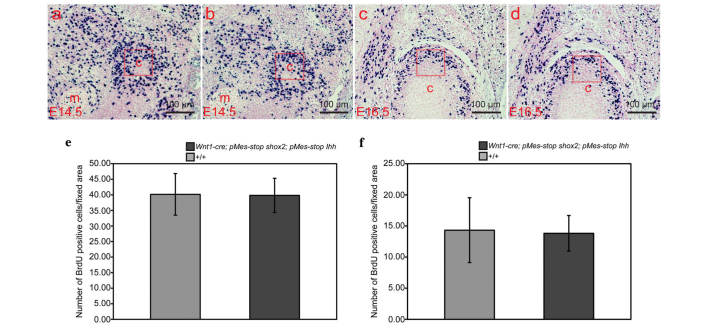
Changes in the cell proliferation in the temporomandibular joints of *Wnt1-Cre*; *pMes-stop shox2*; *pMes-stop Ihh* mice. (a–d) BrdU labeling of cell proliferation in the condylar condensation of wild-type mice and *Wnt1-Cre*; *pMes-stop shox2*; *pMes-stop Ihh* mice at (a and b) E14.5 and (c and d) E16.5. (e and f) Comparison of numbers of BrdU-positive cells in the fixed area of the condylar primoridain between wild-type mice and *Wnt1-Cre*; *pMes-stop shox2*; *pMes-stop Ihh* mice at (e) E14.5 and (f) E16.5. Values are expressed as the mean ± standard deviation (n=3). E, embryonic day; BrdU, bromodeoxyuridine.

The present study further examined whether the overexpression of Ihh was able to decrease the apoptosis that potentially contributed to the TMJ phenotype of the *Wnt1-Cre*; *pMes-stop shox2* mice. The results of the TUNEL assay showed a significantly enhanced number of apoptotic cells in the glenoid fossa of *Wnt1-Cre*; *pMes-stop shox2*; *pMes-stop Ihh* mice compared to that in the wild-type mice at P0 (P<0.05) (Fig. 5a–c), indicating that overexpression of Ihh did not inhibit shox2 overexpression-associated apoptosis of glenoid fossa. In addition, a BrdU labeling assay revealed there was no alteration in the cell proliferation rate in the condyle of the wild-type mice and *Wnt1-Cre*; *pMes-stop shox2*; *pMes-stop Ihh* mice at E14.5 and E16.5 (Fig. 6a–f).

## Discussion

The results of the present study provided solid evidence that Ihh is required for the formation and development of the TMJ. Compared with the results of a previous study by our group (14), the results of the present study demonstrated that overexpres-sion of Ihh rescued the shox2 overexpression-associated reduction of ECM components; however, *shox2*-associated increases of MMP activity, MMP9 and MMP13 levels as well as apoptosis in the TMJ were not sufficiently attenuated, suggesting that overexpression of Ihh partially rescued shox2-associated congenital dysplasia of the TMJ in mice.

In the present study, detailed histological analysis of the TMJ indicated changes in the width of glenoid fossa and condyle between post-natal wild-type mice and *Wnt1-Cre*; *pMes-stop shox2*; *pMes-stop Ihh* mice at P14 and thereafter. The previous study by our group reported that *Wnt1-Cre*; *pMes-stop shox2* mice developed a congenital dysplasia of TMJ, mostly likely attributed to the wasting syndrome, since TMJ is essential for the movement and function of the jaw in mammals (14). To investigate the alterations of the TMJ that may have contributed to the decreases in body weight, a time-course analysis of the body weight was performed in the present study. The body weight was significantly different between wild-type and *Wnt1-Cre*; *pMes-stop shox2*; *pMes-stop Ihh* mice from P14, supporting that alterations of the TMJ may have affected the observed body weight changes.

Ihh is essential for TMJ formation and development, and also for the maintenance of the proper structure and function of the TMJ after it is formed. The expression of Smo, Pct, Gli1, Gli2 and Gli3 as Hh receptors and effector genes accompanies Ihh expression in the process of TMJ development (6,7). It is currently elusive whether the reduction of Ihh expression is a causative factor in the reduction of the ECM and the elevated MMP activity in the TMJ of post-natal *Wnt1-Cre*; *pMes-stop shox2* mice; however, it is consistent with a key role for the Ihh signaling pathway, namely the maintenance of the proper structure and tissue homeostasis of the post-natal TMJ (14). The hypothesis of the present study was that the overexpres-sion of Ihh rescues the TMJ phenotype following upregulation of shox2. To test this hypothesis, *Wnt1-Cre*; *pMes-stop shox2*; *pMes-stop Ihh* mice were bred, in which the overexpression of Ihh in the TMJ was confirmed using immunohistochemical analysis.

To investigate the cellular and molecular alterations contributing to the dysplasia of the TMJ, changes in osteogenic genes, the composition of the ECM, MMPs, apoptosis and proliferation were assessed in a series of developmental stages of *Wnt1-Cre*; *pMes-stop shox2*; *pMes-stop Ihh* mice. During the progression of TMJ development, the transcription factors Runx2 and Sox9 have been implicated in the development of primary cartilage and endochondral ossification (2,18,19). The present study identified that the cell proliferation and the expression of Runx2 and Sox9 were at normal levels in the developing TMJ, indicating upregulation of shox2 and Ihh did not lead to any abnormalities in the progression of TMJ development. To reveal the underlying mechanisms of the dysplasia of the TMJ, the present study then examined changes of ECM composition, MMPs and apoptosis in the TMJ of *Wnt1-Cre*; *pMes-stop shox2*; *pMes-stop Ihh* mice.

Cartilage degradation results from the imbalance between anabolism and catabolism due to increased matrix-degrading proteases and decreased synthesis of matrix, which are largely mediated by the imbalance between anabolic and catabolic cytokine signaling molecules (20,21). These changes are char-acterized by the significant upregulation of MMPs, which are able to cleave aggrecan and collagen, the two most abundant ECM components of articular cartilage (20,22). MMP9 has a central role in connective tissue remodeling and in the turnover of basement membrane (23), while MMP13 has a crucial role in bone formation and remodeling, and is expressed in terminal hypertrophic chondrocytes in the growth plate and in osteoblasts (24,25). MMP9 degrades the aggrecan core protein and denatured type II collagen resulting from initial cleavage by activated MMP1 (26,27). MMP13 degrades fibrillar collagens, including collagen II, and also has the capacity to degrade aggrecan, the hydrodynamically large aggregating proteoglycan of cartilage (28,29). The results of the present study showed that overexpression of Ihh rescued the ECM composition; however, the shox2-associated increases in MMP activity and apoptosis were not attenuated by Ihh overexpres-sion. Overall, these results indicated that overexpression of Ihh was able to partially rescue the shox-associated dysplasia of the TMJ.

In conclusion, the present study showed that overexpression of Ihh partially rescued the shox2 overexpression-associated congenital dysplasia of the TMJ. Further study is required to investigate the association between Ihh and other signals involved in the formation and development of the TMJ. An enhanced understanding of the underlying molecular mechanisms of TMJ organogenesis will aid the improvement of diagnoses and the development of novel therapeutic targets for TMJ disorders.
